# Multifactor Authentication for Smart Emergency Medical Response Transporters

**DOI:** 10.1155/2022/5394942

**Published:** 2022-09-23

**Authors:** Turki Alghamdi, Fayez Gebali, Fares Salem

**Affiliations:** ^1^Faculty of Computer and Information Systems, The Islamic University of Medinah, Prince Naif Ibn Abdulaziz, Al jamiah, Medinah 42351, Saudi Arabia; ^2^Department of Electrical and Computer Engineering, University of Victoria, Victoria, BC, Canada V8W 3P6

## Abstract

Securing telehealth IoT infrastructure is essential to provide high-level medical care and prevent cyberattacks. A vulnerable stage in IoT telehealth is while the patient is being transported to a healthcare facility, the transporter could be an ambulance or an air ambulance. In this paper, we propose a multifactor authentication scheme to secure the system when the patient is in transit to the healthcare facility. We apply this scheme to an ambulance, using physical unclonable functions (PUFs) embedded in the ambulance to facilitate authentication and secure key exchange. We validated the security of the proposed scheme using formal and informal security analysis. The analysis supports our claim that the proposed scheme protects against many types of attacks.

## 1. Introduction

Internet-of-Things (IoT) is becoming essential for infrastructure smart systems such as telehealth, transportation, commerce, and entertainment. This is facilitated through the modern technologies of sensing, telecommunication, high-performance computing, and signal processing. In addition, augmenting IoT technology for computing, artificial intelligence (AI), and machine learning (ML) allows for adding further utility and more system security. These factors are witnessed by the rapid deployment of the technologies of 5G and Wi-Fi 6 communication in IoT systems in the healthcare industry where such systems are controlling valuable infrastructures [1–3]. Providing security to IoT telehealth is mandatory to ensure immunity to attacks [4].

The COVID-19 pandemic accelerated the adoption of telehealth in most of the countries over the world. The healthcare sector in these countries started providing right away most nonurgent clinical services remotely. In response to this and as benefiting from the existence of high technology of IoT and its features of controlling and monitoring of connected smart objects, many systems and edge devices have been proposed and deployed [4, 5].

However, extending IoT telehealth services to emergency medical transport (ambulances and medical evacuation) can provide immediate and responsive healthcare to critical cases while a patient is being transported. Smart ambulances have been proposed to improve the performance of ambulances [6–8]. They use technologies such as IoT, real-time data communication and video streaming, connected vehicles, road traffic monitoring, big data, biomedical sensing, and body area networks to improve the emergency service and minimize response time and provide medical support with the least possible delay [9, 10].

### 1.1. Contributions

The main contributions of this work are as follows:

1. Propose a unified framework for authentication and secure key exchange for emergency response transporters like ambulances or air ambulances
2. Integrate embedded physical unclonable functions (PUF) in the transporter for both context-aware authentication and secure session key exchange

### 1.2. Organization of the Paper

The remainder of this paper is organized as follows: in Section 2, we review the work-related to authentication and secure key exchange for smart emergency medical response systems.  Section 3 provides background on PUFs and their use for context-aware authentication and secure session key exchange. In Section 4, we describe the models and assumptions related to emergency medical response systems and discuss the threats targeting them. In Section 5, the proposed authentication scheme is provided. Formal and informal security analysis and validation of the proposed scheme are provided in Section 6.

### 1.3. Notation and Terms Used

Table 1 summarizes the notations used in this work.

## 2. Related Work

The security of smart ambulances and their infrastructure is mandatory due to the high threats to IoT systems in general and to healthcare systems in particular [8–11]. The smart ambulance system is an essential service and has in its possession valuable information associated with patients and data associated with hospital operations and emergency transporter management. However, such factors attract the attention of cyberattackers for valuable targets [4, 11, 12].

The authors in [13] discussed different types of security threats in smart health systems, including denial of service (DoS) attacks, Fingerprint And Timing-based Snooping (FATS) attacks, router attacks, select and forwarding attacks, sensor attacks, and replay attack. In this paper, categorized attacks with their effectiveness, approach, and security requirements have been analyzed to come up with measurements to secure IoT systems within healthcare environments. It concluded that adopting some security guidelines for system design and development for secure health monitoring is a required action.

El Zouka and Hosni [14] proposed a trust environment model that is responsible for collecting authenticated physiological data from a patient's body. They focused on how to provide reliable, accurate, secure, and real-time patient monitoring data. The authors presented a secure authentication scheme to protect personal health information and guarantee secure communication. The results of this paper show that the suggested scheme achieves a better result than the state-of-the-art authentication mechanisms, as the overhead of the access time was reduced. It mentioned that the key generation time is the highest among the transfer and verification times.

Authors in [4] highlighted the threats, security requirements, challenges, and attacks related to IoT networks. They categorized security threats and challenges as follows:

(1) Generic threats which are applicable on all IoT systems, including hardware vulnerabilities, vulnerabilities of social engineering, legislation challenges, user unawareness, and DoS/DDoS attacks
(2) Architecture layerwise threats: threats at different layers of the IoT architecture are highlighted such as security issues associated with the following layers:
  - Physical/perception layer threats including battery drainage attack, hardware malfunctioning, malign data injection, node cloning, and gaining unauthorized access
  - MAC/adaptation/network layer: collision attack and channel congestion attack are DoS attack types carried out at this level
  - Application layer is where some serious threats can harm this layer including malicious code and weak application security as consequences of weak authentication and authorization mechanism
(3) Security challenges about IoT data, communication, and end applications

Anonymous authentication key agreement scheme was proposed by Yu and Li [15] for multisensor home-based IoT. This scheme was used due to the limited communication and processing capabilities of the edge devices. The authors proposed such a scheme for lightweight authentication and key agreement technology using pairing-based cryptography.

A secure protocol scheme was proposed by Alzahrani [16] to secure a telecare medical information system (TMIS). It employed lightweight symmetric key operations. The proposed scheme was used to establish mutual authentication between patients and physicians in a TMIS-based online system.

Authors in [17] proposed a secure, authenticated, and key agreement scheme in fog-driven IoT healthcare systems. The proposed work in this article was based on the previous secure authenticated and key agreement scheme proposed by [18]. An improvement has been shown in terms of computational time and cost.

**Figure 1:**
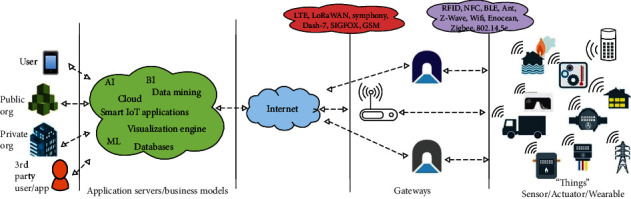
Generic IoT architecture [4].

Generally, an IoT system within any application domain consists of a diversity of entities.  Figure 1 shows the widespread distribution of the devices. However, covering all security issues stated above in such architecture is a very difficult goal. In smart healthcare systems, the hospital and emergency transporter can be thought of as the service providers with which many layered security safeguards can be set up. IoT edge devices, such as sensors and actuators, are very vulnerable since they are decentralized where security levels cannot be guaranteed. The IoT edge devices for smart healthcare control the overall security of the system since they are effectively its weakest link. In this paper, we focus on multifactor authentication as an essential factor for securing smart transporter healthcare. Using physical unclonable functions (PUF) as an embedded means will facilitate authentication and secure session key exchange [19–22].

## 3. Physical Unclonable Function (PUF)

We provide here the necessary background on the organization and operation of PUFs to understand how they can be used and implemented in our proposed system.

PUFs are physical one-way functions that depend on the physical surrounding to operate, and PUFs can be nonelectronic (e.g., magnetic and optical) or electronic (silicon PUF). Silicon PUFs are circuits that are used to extract unique digital fingerprints from electronic chips, and the unique digital fingerprint can be used in many applications like identification of the chip, secret key generation, and multifactor authentication protocols. A silicon PUF is tamper-proof and resists many types of physical and logical attacks [23–28].

**Figure 2:**
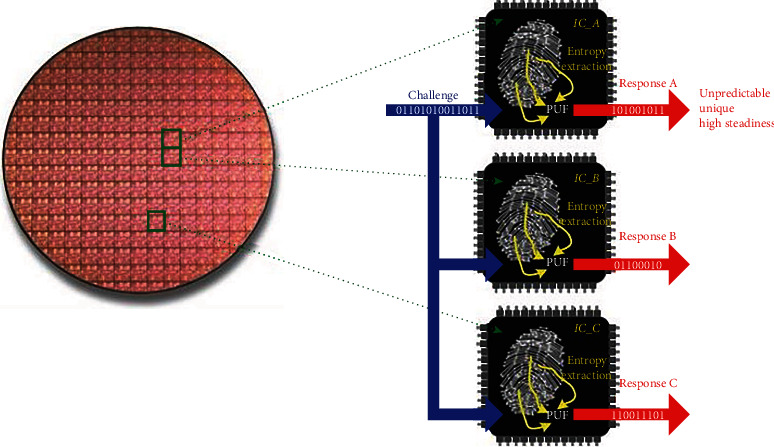
Responses to a challenge of different PUFs on different ICs [29].

The unique ID afforded by the silicon PUF is due to the inevitable random process variations (RPV) inherent in the integrated circuit (IC) manufacturing process, which results in slightly different doping concentration, oxide thickness, and capacitance between different transistors that should be identical on the same chip. Thus, even if all PUF circuits are fabricated based on identical designs and fabrication steps, manufacturing introduces slight variations which give each device its own unique ID as shown in Figure 2. These IDs are unpredictable and are characterized by high steadiness. Any attempt to replicate or disturb the circuit will result in a different response.

Another undesirable effect is the presence of static or slow-varying noise and other dynamic noise sources in each device due to several effects such as

- Transistor shot noise due to the flow of charges across junctions
- Thermal noise due to resistive paths
- Flicker noise due to crystal imperfections

The organization of PUF depends on its type, the most common PUF used is delay-based PUF like ring oscillator (RO) PUF and arbiter PUF and memory-based PUF like static random access memory (SRAM) PUF which can be implemented easily on field programmable gate array (FPGA) that have block random access memories (BRAM) available on the chip.

The authors in [30] reviewed the SRAM, RO, and arbiter PUFs, discussed the statistical models for modeling them, and identified the main parameters defining their performance, and they provided some results showing the performance of these devices and how they depend on the authentication algorithm used.

### 3.1. PUF Operation

PUF operates as part of a challenge-response authentication protocol, where a server presents a challenge to a client (e.g., IoT edge device) having a PUF implemented on its side, which in turn responds to the server with a response that is unique to itself. The client is a device that has a PUF implemented on it, and this could be as part of a microcontroller or microprocessor or on a field programmable gate array (FPGA).

For the client to be authenticated successfully, that response should be valid and the same as what the server expects it to be, as the server already knows the challenge-response pair (CRP) from before [31].

The PUF circuit gives a response at the output when a challenge is applied to the input as follows: *R*[1 : *M* ] = PUF(*C*[1 : *N*]), where *C* [1 : *N*] is the *N*-bit challenge, *R* [1 : *M*] is the *M*-bit response, and PUF (·) is the one-way unique function characterizing the PUF given the physical parameters of a particular IC.

The idea behind CRPs is that at the manufacturing time, the manufacturer builds a database of CRPs for each device (e.g., IoT edge device), the number of CRP pairs depends on the nature of the PUF being employed and the complexity of the circuit design, the manufacturer should make sure to have enough CRPs for the lifetime of the device, or CRP tables would need to be recharged periodically with new CRPs, and this database is to be sent to a trusted certificate authority (CA).

**Figure 3:**
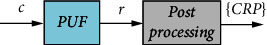
Establishing challenge-response pairs (CRP).

Figure 3 shows how the device manufacturer generates the challenge-response pairs (CRP) for a PUF circuit. The manufacturer issues a set of challenges *c* and applies it to the PUF circuit. The response of the PUF circuit *r* is stored in a dataset. The manufacturer might also do a statistical analysis of the responses, as indicated by the postprocessing block shown in the figure.

The device user can then authenticate the device later when it is deployed in the field by having the server request the CRP from the CA, then query the device with the challenge, and then validate the response it receives back, and the periodicity of authentication differs between applications (e.g., once per day and once per hour). This allows authentication to be done without the need for a secret key to be defined by the manufacturer and to be stored in vulnerable nonvolatile memory (NVM) at the client side since they can be generated by the hardware when needed. The problem with stored keys is that if an attacker was able to get the key, a fake device can be used to have the same secret stored key.

Simple usage of challenge/response pairs (CRP) is not practical because dynamic noise will result in a noisy response that would lead to an unreliable ID and false rejection of the device being authenticated. Furthermore, the noisy response cannot be used to generate a shared session key that is common to both the client and the server.

Eliminating the dynamic noise can be accomplished using two approaches:

1. Using forward error correction (FEC) such as the helper data algorithm [32–34] or fuzzy extractors [35–37]
2. Using statistical properties of the PUF response to generate a reliable response without doing any processing [30, 38, 39]. This approach selects low-noise bits of the response bits such that encoding the PUF response generates noise-free data

### 3.2. Technique of Fuzzy Extractor

Fuzzy extractor is one technique that aims to remove the inevitable noise in the PUF response to facilitate authentication and consistent key agreement between the client and the server [21, 37, 40, 41].

**Figure 4:**
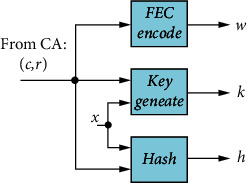
The steps used by the server to obtain helper data *w*, secret session key *k*, and authentication hash *h*.

Figure 4 shows the steps used by the server (hospital) to initiate both server authentication and generate a high-entropy secret key. The server receives the CRP pair from the CA and computes three values: helper data *w*, secret key *k*, and authentication hash *h*. The server uses hashing as a way to use the low-entropy response *r* to generate the stable high-entropy key *k* and the authentication hash *h*. These quantities are obtained based on the CRP and context-aware data *x*. The server will send the challenge *c* and helper data *w* to the client and should never send the response *r* to prevent machine learning attacks on PUFs [21], and it also keeps the secret key *k* and authentication hash *h* stored locally.

**Figure 5:**
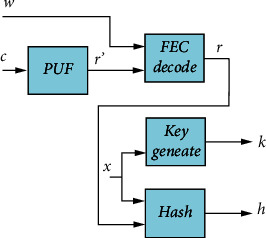
Structure of the system used to obtain secret session key and authentication hash at the client side.

Figure 5 shows the steps used by the client to complete the secret session key exchange and context-aware authentication. The client side receives the challenge *c* and the helper data *w*, and then the PUF produces the actual noisy response *r*′. An estimate of the noise-free response *r* is then produced using the noisy response *r*′ and helper data *w*. The estimated noise-free response *r*, together with the context-aware data *x*, is then used to generate the shared secret session key *k* and context-aware authentication value *h*.

The following equation expresses the key generation process using the fuzzy extractor technique:

(1) $$\begin{array}{c} \left(K_{\left(ed,t\right)},N_{ed}\right)=\text{ke}{\text{y}}_{\text{r}{\text{e}}_{\text{gen}\left(c,r,x\right)}}, \end{array}$$

where *K* (*ed*, *t*) is the secret key, and *N*_ed_ is the secret random number.

The authors in [30] reviewed some of the most recent algorithms that can be used to provide stable authentication and secret key generation without having to use helper data or secure sketch algorithms.

## 4. Preliminaries

In this section, we describe the models and assumptions related to the emergency medical response system.

### 4.1. Smart Emergency Medical Response System

**Figure 6:**
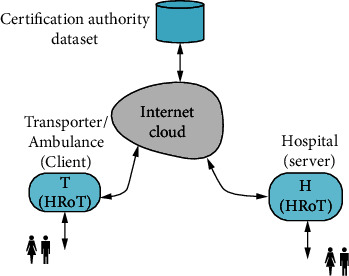
Ambulance-based smart emergency medical response system.

Figure 6 shows the main components of the smart emergency medical response model. The main agents in such a model are as follows:

1. Central healthcare center or server, which is considered to be the hospital (H): doctors and nurses are located in the hospital and need to present their credentials in order to be able to communicate with the remote ambulance. The server could be considered a hardware root-of-trust (HRoT) since it contains tamper-proof security processors and implements layered security protocols
2. Smart emergency response transporter, which is mostly an ambulance but could equally well be a medical helicopter: the transporter is considered the client (T). Paramedics are located in the ambulance and need to present their credentials in order to be able to communicate with the hospital. The ambulance could also be considered a hardware root-of-trust (HRoT) since a tamper-proof physical unclonable function (PUF) is installed in it in order to endow the ambulance with a unique biometric identity (ID). This ID serves as the foundation for authenticating the ambulance and securely generate a shared secret session key common to ambulance and hospital (H) [19–22]
3. Communication medium which could be the internet cloud, a virtual private network (VPN) or a 5G cellular network for increased throughput and reduced latency
4. Certification authority which provides biometrics for certifying

We do not include the patient being transported as a member of the IoT system since the interaction between the patient and the doctor or nurse at the hospital is done through the paramedic in the ambulance.

### 4.2. Adversary Threat Model

Security must be provided for smart emergency medical response systems to ensure data privacy, data integrity, nonrepudiation, and authenticating communicating entities.

We assume the adversary to satisfy the Dolev-Yao model; in addition, the adversary is able to perform the following actions:

1. Obtain the emergency transport vehicle ID through brute force or guessing based on knowledge of the transport vehicle manufacturer and ID sequence assignment
2. Gain access to the computing or communication devices located in the transport vehicle in the field and attempt to extract stored secret keys
3. Launch various attacks to steal the device's secret keys through reading the flash or solid-state drive (SSD) content through fault injection, memory permanence, or cold boot attacks for example
4. Launch a passive attack using side-channel analysis on the edge devices
5. Change the flash or SSD content to run malicious software

### 4.3. Hospital/Server Model

The server side of the smart emergency medical response system under consideration is shown on the right-hand side of Figure 6. The server is located at the healthcare infrastructure at the hospital or healthcare delivery system. It is supported by information technology (IT) personnel and security experts to maintain layered security measures. The computing resources of the server can be assumed limitless. Security is maintained at the application level down to the hardware level. A hardware root-of-trust (HRoT) must be present in order to secure the cryptographic keys and cryptographic primitives and protocols.

The server maintains a virtual private network (VPN) to securely connect to the remote users, who are the health care givers, such as doctors and nurses.

### 4.4. Server Access Device Model

The server access devices allow the emergency practitioner located at the hospital to communicate with the ambulance. These devices could be desktop stations, laptops, mobile phones, or even virtual reality (VR) devices. These devices allow the emergency practitioners to extract the data of the edge devices and monitor the status of the patient. Several security attacks such as reverse engineering, SQL and object injection, and phishing could be launched due to the vulnerabilities of these access devices.

### 4.5. Transporter/Client Model

The client side of the smart emergency medical response system under consideration is shown on the left-hand side of Figure 6. The client is the emergency transport vehicle which could be an ambulance or medical evacuation helicopter. Emergency practitioners located at the transporter are typically paramedics that use different types of IoT edge devices to monitor the patients being transported. The computing resources at the transporter must be able to implement layered security starting from the applications down to the hardware processors. A hardware root-of-trust (HRoT) could be implemented in order to secure the cryptographic keys and cryptographic primitives and protocols.

### 4.6. Transporter Model

The transporter in our proposed scheme acts as a gateway that is located at the ambulance vehicle, medical helicopter, ambulance control center, hospital, or any geographically remote area. In the transporter device, secured layers must be implemented by starting from the applications down to the hardware processors. In order to secure the cryptographic keys and cryptographic primitives and protocols, a hardware root-of-trust (HRoT) would be implemented.

A random number (nonce) *N*_ed_ is generated at the gateway and applied to a cryptographic hash function to generate the secret key *K*_(ed, *t*)_ with a predetermined number of bits and high entropy. This serves two purposes: *N*_ed_ serves to query the presence and freshness of the connection with the IoT device, and *K*_(ed, *t*)_ serves to generate a one-time password (OTP) for use in cryptographic operations for the current session. The key generation using the fuzzy extractor process can be expressed by the following equation:

(2) $$\begin{array}{c} \left(K_{\left(ed,t\right)},r\right)=\text{ke}{\text{y}}_{\text{gen}\left(\text{I}{\text{D}}_{\text{ed}},c,N_{\text{ed}}\right)}, \end{array}$$

where *K*_(ed, *t*)_ is the secret key, and *r* is the helper data. The authentication process starts by the transporter to generate the quantities *N*_ed_, *K*_(ed, *t*)_, and *r*.

The authenticating device also queries the dataset associated with the device using its identity ID_ed_ and a selected challenge *c* to obtain a response *w*. *N*_ed_ is then encoded using a linear block code such as BCH and the resulting redundant bits are XORed with the PUF response to generate the helper data *r*. The helper data is public and is transmitted, along with *c*, to the IoT edge device at the start of a session.

At the start of a session, the transporter computes a nonce *N*_ed_ and chooses a challenge *c* to generate a one-time password/secret key *K*_(ed, *t*)_ according to Eq. (2). The gateway sends the chosen challenge and helper data to the edge device, and this can be expressed by

(3) $$\begin{array}{c} T⟶Ed:Hd=\left(c,r,N_{\text{ed}}\right). \end{array}$$

The edge device is capable of generating its own copy of *K*_(ed, *t*)_ through the publicly received quantities *c* and *r*. At this stage, both the transporter and edge device know *K*_(ed, *t*)_ and *N*_ed_ based on Eq. (1).

### 4.7. Edge Device Model

The IoT edge devices are used for remote smart emergency precheck of the patient while being transferred to the hospital. They are based on embedded systems where secret keys used for cryptographic primitives are PUF-based as discussed in Section 3. Using a PUF ensures that each device has a unique ID and a session secret key that is generated by the hardware and not stored in vulnerable nonvolatile memory [24, 25, 37, 42, 43]. These devices are authenticated essentially based on a challenge-response pair (CRP). A challenge *c* is issued by the server where the expected response *w* is generated. The edge device as a client receives a challenge *c* and generates a noisy response *r*′. Authors in [19, 21, 35, 41] highlighted a description of how the noisy data of a PUF can be used to generate a consistent session key to be used for authentication and secure message exchange.

### 4.8. Communication Model

The communication model in the smart emergency medical response system is comprised of the following main components:

- Secure server *S* connecting devices to the Internet. The server could be considered a hardware root-of-trust (HRoT) since it contains tamper-proof security processors and implements layered security protocols
- Transporter *T* connecting the edge devices to the Internet. The transporter could be considered a hardware root-of-trust (HRoT) since there is one transporter in each remote location; so, it would not impose too much expense on the system deployment in relation to the security benefits dividends. *T* contains tamper-proof security processors and implements layered security protocols also
- Internet connection (*cloud*) which can rely on 5G or Wi-Fi 6 technologies for fast data throughput and less latency
- Handheld devices (Hd) which are used by the emergency practitioners to access the system through the server. The handheld devices are located where emergency practitioners practice their job at ambulances, hospitals, research centers, etc. The handheld devices could be connected to the server through secure virtual private networks (VPN) to reduce any possible attacks
- IoT edge devices (Ed) which are typically located at the ambulance transporter, or emergency rooms, and are connected through a local-area network. These devices typically have limited computation capabilities and a small memory footprint

### 4.9. Multifactor Authentication

Traditional authentication aims at providing access control through the use of a user or device identity (ID) and a password. Once the password is inferred or stolen, security of the system is destroyed. For this reason, multifactor authentication is used to enhance security. Multifactor authentication uses one or more of the following:

1. What you know (e.g., password or passphrase)
2. What you have (e.g., smart card)
3. What you are (e.g., biometrics)
4. Your context (e.g., location)

For the purposes of this work, multifactor authentication used for medical personnel uses the medical personnel biometrics in addition to sending a one-time password or a nonce to the mobile device known to be associated with each person.

Multifactor authentication is used for the hardware of the emergency response system through the use of physical unclonable functions (PUFs), as discussed in Section 3. Each device is associated with a unique device ID (analogous to a user ID), in addition to a secret key that is generated or derived from the PUF response based on using a fuzzy extractor as discussed in Section 3.2 or other noise-removing techniques.

## 5. The Proposed Authentication Scheme

The proposed authentication protocol is divided into four phases as follows:

1. Predeployment
2. Registration
3. Login
4. Authentication

The transporter (*T*) manages all communications with the server/hospital (*S*). Similarly, the server (*S*) manages all communications with the transporter (*T*).

### 5.1. Predeployment

This phase considers the predeployment steps needed for our proposed authentication protocol in a smart emergency response transporter system.

#### 5.1.1. Server

Secured communication is established by the server with the registration authority (RA) using a symmetric secure key *Ks* where a unique ID*_s_* is assigned to the server. Since the server can be considered a hardware root-of-trust (HRoT), several layers of security protocols are implemented. Credentials for the transporter, handheld devices, and edge devices are obtained throughout the server communication with the RA. As a result, this process comprises the main components of our proposed system.

#### 5.1.2. Transporter

The transporter is assigned a symmetric secret key *Kts* and a unique identity ID*_t_* in order to communicate with the server.

#### 5.1.3. Handheld Devices

The handheld device is used by the emergency practitioner as a channel of access in order to communicate with IoT edge devices, hospitals, and emergency centers through either the server or the transporter. Each handheld device is assigned a symmetric secret key *K*_*hds*_ and a unique identity ID*_Hd_*. The user will be requested to provide a password PW_hd_ and a biometric *B*_*hd*_ prior to using the handheld device.

#### 5.1.4. Edge Device

The edge device is manufactured with a unique ID (ID_ed_). However, the manufacturer generates a CRP dataset of the device after fabricating it. The CRP dataset is shared only with an authenticating entity. The helper data, stated in Section 3.2, is included by the fabricator.

### 5.2. Registration Phase

**Figure 7:**
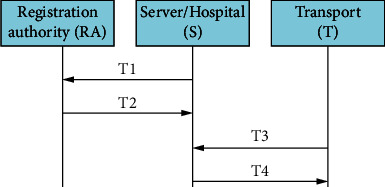
Registration phase of the emergency medical response system.

The server, device fabricator, and RA are the main entities of the smart emergency medical response system. They are responsible for manufacturing the transporter, handheld devices, and edge devices. Secure communication with RA is established by the server and the device fabricator using public key infrastructure (PKI). The registration phase is illustrated in Figure 7 showing how the manufacturer implements this phase for the transporter, edge devices, and handheld devices. Of course, any communication channel is prone to transmission errors. Hence, it is implicitly assumed that error control coding and protocols are implemented. The transactions taking place in this phase are as follows:

- T1: the server sends a request to the RA to gain the data associated with the transporter, handheld devices, and edge devices
- T2: the RA sends to the server the requested data
- T3: the transporter sends a request to the server to gain the data associated with edge devices connected to it
- T4: the server sends to the transporter the requested data

In order to reduce the chance of attacks, we suppose in our proposed protocol that the connection between the RA and the transporter, handheld devices, or edge devices are only allowed through the server.

#### 5.2.1. Transporter Registration

The server initiates the registration of the transporter by communicating with the RA to request and obtain the transporter secret key *K*_*ts*_. The server *S* sends a challenge message *m*; thus, a response *r* is sent back by the RA, and this can be demonstrated by the following equations:

(4) $$\begin{array}{c} S:m_{t1}=E\left(K_{sr},\left(\text{I}{\text{D}}_{s}\left\|\text{I}{\text{D}}_{t}\right\|N_{s1}\right)\right), \end{array}$$

where a challenge for RA as an encrypted message that includes a nonce *N*_*s*1_ is prepared by the server.

(5) $$\begin{array}{c} S⟶\text{RA}:\text{Request}\left(\text{I}{\text{D}}_{s},\text{I}{\text{D}}_{\text{ra}},m_{t1}\right). \end{array}$$

The operation in this equation defines how the server sends a request to communicate with RA.

(6) $$\begin{array}{c} \text{RA}:Vt=h\left(\text{I}{\text{D}}_{s}\left\|\text{I}{\text{D}}_{t}\right\|{\text{K}}_{ts}\left\|N_{s1}\right.\right), \end{array}$$

where the RA prepares the hash *V*_*t*_ to be used later for authentication between *S* and *T*. It should be mentioned that these hash values or values are stored by the devices in temporary storage, and they get regenerated at the start of each session.

(7) $$\begin{array}{c} \text{RA}⟶S:E\left(K_{\text{sr}},\left(K_{\text{ts}}\left\|\text{I}{\text{D}}_{t}\right\|V_{t}\left\|N_{s1}\right.\right)\right). \end{array}$$

As shown here, the nonce *N*_*s*1_, as a proof of existence and freshness, is sent back by the RA.

(8) $$\begin{array}{c} S:m_{t2}=E\left(K_{ts},\left(\text{I}{\text{D}}_{t}\left\|V_{t}\right\|N_{s2}\right)\right). \end{array}$$

The operation in equation (8) shows how the server prepares a challenge for *T* as an encrypted message that includes the nonce *N*_*s*2_.

(9) $$\begin{array}{c} S⟶T:\text{Request}\left(\text{I}{\text{D}}_{s},\text{I}{\text{D}}_{t},m_{t2}\right), \end{array}$$

where a request is sent by the server to communicate with T.

(10) $$\begin{array}{c} T⟶S:E\left(K_{ts},N_{s2}\right). \end{array}$$

The operation in Eq. (10) represents how the transport responds to the challenge by sending back the encrypted nonce *N*_*s*2_.

Nonces *N*_*s*1_ and *N*_*s*2_ are defined to ensure message freshness and the hash *V*_*t*_. Therefore, they will be used later in the authentication phase between *S* and *T*.

#### 5.2.2. Registration of the Edge Device

The registration of the edge device is initiated after defining all the system entities to the server. This includes all edge devices connected to the transporter. The server communicates with the RA to request the secret key of each edge device (*K*_ed_) and CRP_ed_ dataset.

(11) $$\begin{array}{c} S:m1=E\left(K_{\text{sr}},\left(\text{I}{\text{D}}_{s}\left\|\text{I}{\text{D}}_{t}\right\|\text{I}{\text{D}}_{\text{ed}}\left\|N_{s1}\right.\right)\right). \end{array}$$

The server sends a request to communicate with RA as defined by the following equation:

(12) $$\begin{array}{c} S⟶\text{RA}:\text{Request}\left(\text{I}{\text{D}}_{s},\text{I}{\text{D}}_{\text{ra}},m_{1}\right), \end{array}$$

where a challenge for RA as an encrypted message that includes a nonce *N*_*s*1_ is prepared by the server.

(13) $$\begin{array}{c} RA:V_{d}=h\left(\text{I}{\text{D}}_{\text{ed}}\left\|K_{\text{ed}t}\right\|N_{s1}\right), \end{array}$$

where the hash *V*_*d*_ is prepared by the RA to be used later for authentication between S and T.

(14) $$\begin{array}{c} \text{RA}:m_{2}={\text{TID}}_{\text{ed}}\left|\left|T\right|\right|H\left|\left|{\text{CRP}}_{\text{ed}}\right|\right|\left(n,k,d\right)\left|\left|V_{\text{ed}}\right|\right|N_{s1}. \end{array}$$

As shown above at Eq. (14), the nonce *N*_*s*1_ is sent back by the RA.

(15) $$\begin{array}{c} \text{RA}⟶S:E\left(K_{sr},m_{2}\right), \end{array}$$

(16) $$\begin{array}{c} S:m_{3}=E\left(K_{\text{ts}},\left({\text{ID}}_{\text{ed}}\left|\left|V_{\text{ed}}\right|\right|N_{s2}\right)\right). \end{array}$$

The operation in Eq. (16) shows how the server prepares a challenge for Ed as an encrypted message that includes the nonce *N*_*s*2_.

(17) $$\begin{array}{c} S⟶T:\text{Request}\left({\text{ID}}_{\text{s}},\text{I}{\text{D}}_{t},m_{3}\right), \end{array}$$

where a request is sent by the server to communicate with *T*.

(18) $$\begin{array}{c} T⟶S:E\left(K_{ts},N_{s2}\right). \end{array}$$

The operation in Eq. (18) represents how the transport responds to the challenge by sending back the encrypted nonce *N*_*s*2_.

(19) $$\begin{array}{c} T:m_{4}=E\left(K_{\text{edt}},\left({\text{ID}}_{\text{ed}}\left|\left|V_{\text{ed}}\right|\right|N_{t}\right)\right), \end{array}$$

(20) $$\begin{array}{c} T⟶E:\text{Request}\left({\text{ID}}_{t},\text{I}{\text{D}}_{\text{ed}},m_{4}\right), \end{array}$$

(21) $$\begin{array}{c} \text{Ed}⟶T:E\left(K_{\text{edt}},N_{t}\right). \end{array}$$

By looking at operations in Eqs. (19), (20), and (21), (*K*_*edt*_) and limited attempts of login are enforced where the first login is successful when *V*_*hd*_ matches *V*′; however, CRP_ed_ dataset is obtained in correspondence to the built-in PUF and fuzzy extractor data (i.e., BCH code with parameters (*n*, *k*, *d*)) and the generator matrix (*G*) and parity check matrix (*H*). During the communication between the transporter and the edge device, the hash *V*_*ed*_ and TID_ed_ will be used for the duration of the session.

#### 5.2.3. Registration of Handheld Device

When the handheld device Hd communicates with the server *S*, the registration is initiated and, in turn, *S* communicates with RA to obtain the identity of the handheld device (ID_hd_). The password PW_hd_ selected by the user is passed to the RA by the server *S*. The operations in Eqs. (22)–(29) are similar to those in the registration of the edge device.

(22) $$\begin{array}{c} \text{Hd}:{\text{hd}}_{m1}=E\left(K_{\text{hds}},\left({\text{ID}}_{\text{hd}}\left\|N_{\text{hd}}\right.\right)\right), \end{array}$$

(23) $$\begin{array}{c} \text{Hd}⟶S:\text{Request}\left({\text{ID}}_{\text{hd}},\text{I}{\text{D}}_{s},\text{h}{\text{d}}_{m1}\right), \end{array}$$

(24) $$\begin{array}{c} S:{\text{hd}}_{m2}=E\left(K_{\text{sr}},\left({\text{ID}}_{s}\left|\left|{\text{ID}}_{\text{hd}}\right|\right|N_{s1}\right)\right), \end{array}$$

(25) $$\begin{array}{c} S⟶\text{RA}:\text{Request}\left({\text{ID}}_{s},\text{I}{\text{D}}_{\text{ra}},m_{2}\right), \end{array}$$

(26) $$\begin{array}{c} \text{RA}:V_{\text{hd}}=h\left(\left.{\text{ID}}_{\text{hd}}\right\|{\text{PW}}_{\text{hd}}\right), \end{array}$$

(27) $$\begin{array}{c} \text{RA}⟶S:E\left(K_{sr},\left({\text{ID}}_{\text{hd}}\left|\left|V_{\text{hd}}\right|\right|N_{s1}\right)\right), \end{array}$$

(28) $$\begin{array}{c} S⟶\text{Hd}:E\left(K_{\text{hds}},\left({\text{ID}}_{\text{hd}}\left|\left|V_{\text{hd}}\right|\right|N_{s2}\right)\left\|{\text{hd}}_{m1}\right.\right), \end{array}$$

(29) $$\begin{array}{c} \text{Hd}⟶S:E\left(K_{\text{hds}},N_{s2}\right), \end{array}$$

where the hash *V*_*hd*_ will be used later in the authentication phase between *S* and Hd.

### 5.3. Login Phase

The operation in this phase starts with the emergency practitioner who signs in to the system using a handheld device. The authentication process will be based on three factors: the device *ID* (*ID_hd_*), the password, and the biometric of the emergency practitioner.

(30) $$\begin{array}{c} \text{Hd}:V_{\text{hd}}^{\prime }=h\left(\text{IDhd}\left\|{\text{PW}}_{\text{hd}}\right.\right). \end{array}$$

The handheld computes and sends *V*_hd_′ to the server.

(31) $$\begin{array}{c} S:V_{\text{hd}}==V_{\text{hd}}^{\prime }. \end{array}$$

Limited attempts of login are enforced where the first login is successful when *V*_*hd*_ matches *V*_*hd*_′; however, if it is not successful, then another attempt is allowed by using more authentication factor such as answering the security question. In case of exceeding login attempts, then the device terminates the login operation, and the user will be requested to register again.

### 5.4. Authentication Phase

In the smart emergency medical response system, the emergency practitioner will need to communicate with the edge devices within the ambulance; therefore, mutual authentication is required in order to establish secure communication between the system entities. The purpose of mutual authentication in this phase is to let all entities of the system verify each other. Therefore, authentication will be required in both directions, forward and backward communication (e.g., handheld device to server and server to handheld device). In this case, we consider four entities to be involved in the authentication phase which are the server *S*, the transporter *T*, the handheld device Hd, and the edge device Ed. The authentication phase goes through three stages as follows:

1. Handheld device to server stage
2. Server to transporter stage
3. Transporter to Edge device stage

#### 5.4.1. Handheld Device to Server Stage

This stage is started by the handheld device choosing a nonce *N*_1_ where its current location is obtained, and a dynamic identity is calculated. However, to preserve anonymity and untraceability for each session, a unique dynamic identity is generated depending on the nonce *N*_1_.

(32) $$\begin{array}{c} {\text{DID}}_{\text{hd}}={\text{ID}}_{\text{hd}}⊕N_{1},\\ \text{Hd}\_S=\left({\text{DID}}_{\text{hd}}\left\|N_{1}\right\|L_{\text{hd}}\right)⊕V_{\text{hd}}, \end{array}$$

where *V*_*hd*_ was obtained from RA in Eq. (26).

To calculate the quantities, the handheld device applies hash chains as follows:

(33) $$\begin{array}{c} H_{\text{hd}s}=h\left({\text{DID}}_{\text{hd}}\left\|N_{1}\right\|L_{\text{hd}}\right). \end{array}$$

A massage is sent by the handheld device to the server by performing the following equation:

(34) $$\begin{array}{c} \text{Hd}⟶S:\left(Hd\_S,H_{hd\_s}\right), \end{array}$$

where server *S* assures the presence and the freshness of the Hd. The message in this equation is received by the server which computes Hd_S using the stored value *V*_*hd*_ (see Eq. (35)).

(35) $$\begin{array}{c} {\text{DID}}_{\text{hd}}\left\|N_{1}\right\|L_{\text{hd}}=Hd\;S⊕V_{\text{hd}}. \end{array}$$

To prevent attacked reply and computes *ID*_∗_*hd*__ and *H*_*hd*_*s*_^∗^, the freshness of received nonce *N*_1_ is checked by the server as follows:

(36) $$\begin{array}{c} {\text{ID}}_{\text{hd}}^{*}={\text{DID}}_{\text{hd}}⊕N_{1},\\ H_{hd\_s}^{*}=h\left({\text{DID}}_{\text{hd}}\left|\left|N_{1}\right|\right|L_{hd}\right). \end{array}$$

The authentication of the handheld device is successful if the following conditions are met: *ID*_*hd*_^∗^ = = *ID*_*hd*_, *H*_*hd*_*s*_^∗^ = = *H*_*hd*_*s*_, and *L*_*hd*_^∗^ ≤ *L*_*hd*−1_ + Δ where ∆ is the maximum change that is allowed in the location between two sessions. This verifies the integrity of the message; otherwise, the server will terminate the session with the handheld device.

#### 5.4.2. Server to Transporter Stage

In this stage, a message is prepared by server *S* to be sent to the transporter *T*. The process starts by generating a nonce *N*_2_ where a dynamic identity of the server DID*_s_* is computed. This can be shown as follows:

(37) $$\begin{array}{c} {\text{DID}}_{s}={\text{ID}}_{s}⊕N_{2},\\ \text{ST}=\left({\text{DID}}_{s}\left|\left|N_{1}\right|\right|N_{2}\right)⊕V_{t},\\ H_{st}=h\left({\text{DID}}_{s}\left|\left|N_{1}\right|\right|N_{2}\right). \end{array}$$

The message is sent by the server to the transporter as follows:

(38) $$\begin{array}{c} S⟶T:\left(ST,H_{st}\right). \end{array}$$

Then, the message in Eq. (38) is received by the transporter which computes ST using the stored value *V*_*t*_:

(39) $$\begin{array}{c} {\text{DID}}_{s}\left|\left|N_{1}\right|\right|N_{2}=\text{ST}⊕V_{t}, \end{array}$$

(40) $$\begin{array}{c} {{\text{ID}}^{*}}_{s}={\text{DID}}_{s}⊕N_{2}, \end{array}$$

(41) $$\begin{array}{c} H_{\overset{*}{st}}=h\left({\text{DID}}_{s}\left|\left|N_{1}\right|\right|N_{2}\right). \end{array}$$

*ID* ^∗^_*s*_ in Eq. (40) and $H_{\overset{*}{st}}$ in Eq. (41) is computed by the transporter. It compares $H_{\overset{*}{st}}$ with the received value *H*_*st*_ where the integrity of the message is verified; however, in case it is not verified, then the session between the server and transporter is terminated by the transporter.

#### 5.4.3. Transporter to Edge Device Stage

A massage is prepared by the transporter *T* to be sent to the edge device Ed. The process starts by generating a nonce *N*_3_.

(42) $$\begin{array}{c} T\_Ed=\left(N_{1}\left|\left|N_{2}\right|\right|N_{3}\right)⊕V_{Ed},\\ H_{t\_ed}=h\left(N_{1}\left|\left|N_{2}\right|\right|N_{3}\right). \end{array}$$

The following shows that the transporter sends a message to the edge device:

(43) $$\begin{array}{c} T⟶Ed:\left(T\_Ed,H_{t\_ed}\right) \end{array}$$

Afterward, when the message is received from the transporter in Eq. (43), the following equation shows the transporter in Eq. (43), and the following equation shows the transporter computes T_Ed using the stored value *v*_*ed*_:

(44) $$\begin{array}{c} N_{1}\left|\left|N_{2}\right|\right|N_{3}=T\;Ed⊕V_{ed},\\ H_{t\_ed}^{*}=h\left(N_{1}\left|\left|N_{2}\right|\right|N_{3}\right), \end{array}$$

where *H*_*t*_*ed*_^∗^ is computed by the edge device Ed.

#### 5.4.4. The Backward Process

The following shows the reverse path for the stage of (transporter to edge device) where the edge devices start by generating a nonce *N*_4_ and computing its dynamic identity DID_ed_ and the session key SK.

(45) $$\begin{array}{c} {\text{DID}}_{\text{ed}}={\text{ID}}_{\text{ed}}⊕N_{4}, \end{array}$$

(46) $$\begin{array}{c} \text{SK}=h\left(N_{1}\left\|N_{2}\right\|N_{3}\left\|N_{4}\right.\right), \end{array}$$

(47) $$\begin{array}{c} D_{ed}=\left.{\text{ID}}_{\text{edi}}\right|i\in D, \end{array}$$

where *D*_*ed*_ represents the set of identities of all edge devices seen by the particular edge device being authenticated, and Ed is the set of all edge devices in the IoT network.

A reply is prepared by the edge device to be sent to the transporter by computing the following quantities:

(48) $$\begin{array}{c} \text{Ed}\_T=\left({\text{DID}}_{\text{ed}}\left|\left|D_{ed}\right|\right|N_{4}\right)⊕V_{ed}, \end{array}$$

(49) $$\begin{array}{c} H_{\text{ed}\_t}=h\left(N_{4}\left\|\text{SK}\right.\right). \end{array}$$

Afterward, a message is sent by the edge device to the transporter as follows:

(50) $$\begin{array}{c} \text{Ed}⟶T:\left(\text{Ed}\_T,H_{\text{ed}\_t}\right). \end{array}$$

When a message is received by the transporter, the transporter extracts *N*_4_ and D*_ed_*:

(51) $$\begin{array}{c} \left({\text{DID}}_{\text{ed}}\left|\left|D_{ed}\right|\right|N_{4}\right)=\text{Ed}\_T⊕V_{t}. \end{array}$$

The following shows that the identity of the edge device is computed by the transporter:

(52) $$\begin{array}{c} {\text{ID}}_{\text{ed}}={\text{DID}}_{\text{ed}}⊕N4. \end{array}$$

The authentication of the edge device is successful when the following conditions are satisfied: *ID*^∗^_*ed*_ = = *ID*_*ed*_, Ded=ϕ, D_*ed*_ ⊂ D, and *H*^∗^_*ed*_*t*_ = = *H*_*ed*_*t*_, when getting the value *N*_4_, the message integrity is verified by the transporter using Eq. (49) and, therefore, ensures the validity of the above equations. Once the transporter got the value *N*_4_, it independently calculates its dynamic identity and the session key SK using Eq. (46).

(53) $$\begin{array}{c} {\text{DID}}_{t}={\text{ID}}_{t}⊕N_{3}. \end{array}$$

The following shows how the transporter embeds the values *N*_3_ and *N*_4_:

(54) $$\begin{array}{c} \text{TS}=\left({\text{DID}}_{t}\left|\left|N_{3}\right|\right|N_{4}\right)⊕V_{t}. \end{array}$$

And computes *H*_*ts*_ as follows:

(55) $$\begin{array}{c} H_{ts}=h\left(N_{3}\left|\left|N_{4}\right|\right|\text{SK}\right). \end{array}$$

Afterward, a message will be sent to the server by the transporter:

(56) $$\begin{array}{c} T⟶S:\left(TS\left\|H_{ts}\right.\right). \end{array}$$

Once a message is received by the server, *N*_3_ and *N*_4_ will be extracted as follows:

(57) $$\begin{array}{c} \left({\text{DID}}_{t}\left|\left|N_{3}\right|\right|N_{4}\right)=\text{TS}⊕V_{t}. \end{array}$$

Once the server received the values *N*_3_ and *N*_4_, it independently calculates its dynamic identity and the session key SK using Eq. (46).

The message integrity is verified by the server by calculating *H*^∗^ using Eq. (55) and, therefore, compares it with the received *H*_*ts*_.

The next step for the server is to embed the values *N*_2_, *N*_3_, and *N*_4_ by the following quantity:

(58) $$\begin{array}{c} S\;Hd=\left(N_{2}\left|\left|N_{3}\right|\right|N_{4}\right)⊕V_{hd}, \end{array}$$

(59) $$\begin{array}{c} H_{shd}=h\left(N_{2}\left|\left|N_{3}\right|\right|N_{4}\right). \end{array}$$

At this stage, a message will be sent to the handheld device:

(60) $$\begin{array}{c} S⟶Hd:\left(S\;Hd\left\|H_{s\_hd}\right.\right). \end{array}$$

Once a message is received by the handheld device, *N*_2_, *N*_3_, and *N*_4_ will be extracted as follows:

(61) $$\begin{array}{c} \left(N_{2}\left|\left|N_{3}\right|\right|N_{4}\right)=S\;Hd⊕V_{hd}. \end{array}$$

Using the received values (*N*_2_, *N*_3_, and *N*_4_), the handheld device, independently, calculates its dynamic identity and the session key SK using Eq. (46).

The message integrity is verified by the handheld device by calculating $H_{\overset{*}{s\_hd}}$ using Eq. (59) and, therefore, compares it with the received *H*_*s*_*hd*_.

## 6. Security Analysis and Validation of the Proposed Scheme

Showing the strength of the proposed scheme, formal and informal security analyses are conducted to analyze our proposed protocol. In this section, we present the inferences obtained from the analysis.

### 6.1. Formal Analysis

AVISPA (Automated Validation of Internet Security Protocols and Applications) tool is used here to verify the robustness of the proposed protocol. AVISPA is a formal protocol verification tool that can be used to build, analyze, and validate the security properties of network security protocols. Four different verification back-end tools are integrated by AVISPA to implement a variety of approaches to analyze the protocols: OFMC (On-the-fly Model-Checker), CL-AtSe (Constraint-Logic-based Attack Searcher), SATMC (SAT-based Model-Checker), and TA4SP (Tree Automata-based Protocol Analyser) [44–48]. In order to analyze our protocol in AVISPA, we modeled it in a modular and formal language called High-Level Protocol Specification Language (HLPSL). The semantic and syntactic correctness of HLPSL modeling can be validated using the SPAN (Security Protocol ANimator) tool which provides a graphical interface that helps to debug the HLPSL specification [49].

**Figure 8:**
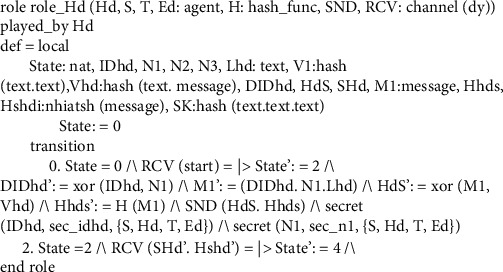
Handheld device role specifications.

**Figure 9:**
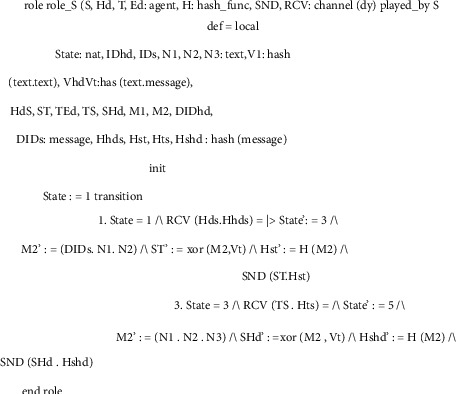
Server (*S*) role specifications.

**Figure 10:**
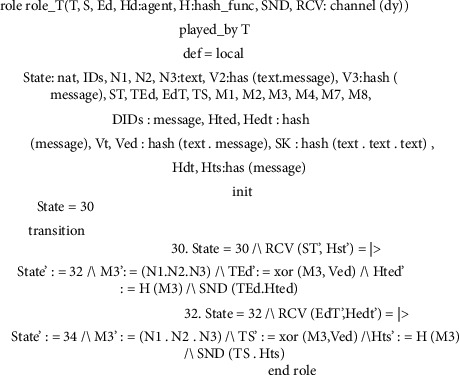
Transporter role specifications.

**Figure 11:**
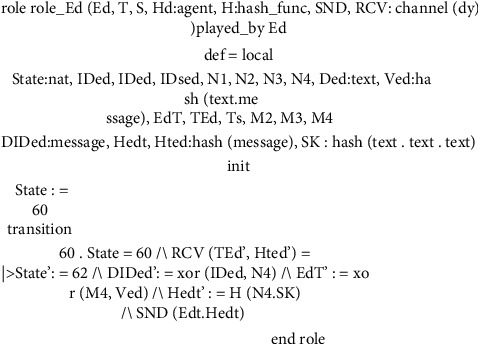
Edge device role specifications.

**Figure 12:**
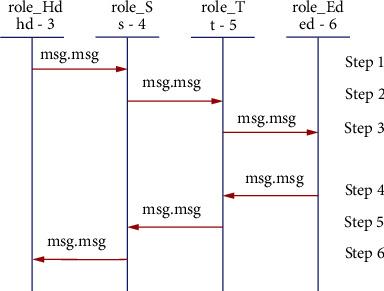
Smart emergency medical response system protocol simulation on SPAN.

This subsection explores several roles for system entities, the session, the goal, and the environment of the proposed scheme. In Figures 8–11, we illustrate HLPSL code for our proposed scheme. These figures show the HLPSL language code that defines the configuration of the sessions, the environment, and the security goals to be achieved by our proposed scheme. The figures also show the definitions of the security goals declared to be secrets in the entity's functions and the values that are authenticated by the entities. As mentioned above in Section 5.4, the communication between the system entities goes forward through three stages (stage 1: handheld device to server, stage 2: server to transporter, and stage 3: transporter to edge device) and backward in the reverse direction. The role for handheld device Hd is implemented in Figure 8 where a secret message is sent to the server *S* as stage 1. In Figure 9, we demonstrate how we have implemented the role of the server *S* where the message is received and then passed to the transporter *T* (i.e., stage 2). As depicted in Figure 10, the implemented role of transporter *T* shows the third stage which passes the message to edge device Ed. The role of edge device Ed is implemented in Figure 11 where a secret message is received and the backward direction start.  Figure 12 shows the protocol execution using SPAN software, where all agents exchange the messages in the authentication phase.

**Figure 13:**
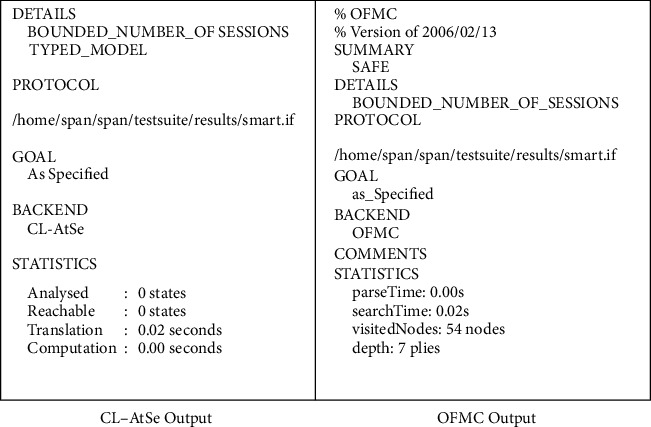
The results using OFMC and CL-AtSe backends.

In conclusion, the results are shown in Figure 13 clearly. The strength of the proposed scheme against attacks is tested using the OFMC backend. Figure 13 demonstrates that our protocol can resist critical attacks and is declared safe to use in the smart emergency medical response system. Similarly, the CL-AtSe backend also declared the protocol as safe. Consequently, it has been shown that the proposed scheme is immune to man-in-the-middle and replay attacks.

### 6.2. Informal Analysis

In this section, we present the robustness of our proposed protocol against several known attack.

#### 6.2.1. Prevention against Reply Attack

When the network traffic is captured by an unauthorized party, a replay attack occurs where the traffic of the network is maliciously delayed or repeated while impersonating the legitimate agent. The random method is used to resist a replay attack including the nonces *N* which is changed in each session can prevent such type of attack.

#### 6.2.2. Prevention against Impersonation Attack

Identity theft is called impersonation attack which leads to the disclosure of information to a nonlegitimate agent. When an attacking attempt by (for instance) Bob to impersonate an emergency professional, this attempt cannot succeed because the password or the biometric will be required for three-factor authentication to access the handheld device.

#### 6.2.3. Prevention against MITM Attack

In MIMT (Man-In-The-Middle) attack, the information exchanged between the two legitimate parties is intercepted by an intruder who also breaks virtually their connection. However, our proposed scheme offers mutual authentication where the transmitted messages are further protected by the secret values and nonces. Without knowing those secret values, it is not possible for anyone to forge legally authenticated messages. Therefore, the MITM attack is prevented by the proposed scheme.

#### 6.2.4. Prevention against Confidentiality/Privacy Attack

In our proposed scheme, the messages between Hd, *S*, *T*, and Ed can be intercepted by the adversary since all messages are sent in plain text. The confidential data cannot be accessed by an attacker through messages because the confidential data is protected by secret parameters shared securely between the entities (e.g., *V*_*hd*_ and *V*_*t*_). Furthermore, the confidential data is shielded by a one-way hash function and bitwise XOR operator. Therefore, the transmitted parameters cannot be unfolded and secured information cannot be extracted.

#### 6.2.5. Prevention against SK Guessing Attack

Communication parties, namely, Hd, *S*, *T*, and Ed along with four randomly selected nonces, construct the session key. Security property relies on the randomness of the input values, which prevents attacking through SK when an attacker tries to guess it. The likelihood of guessing the right key SK by an attacker is tenuous, provided that nonces *N*_1_, *N*_1_, *N*_1_, and *N*_1_ are chosen randomly in every session.

#### 6.2.6. Location-Based Authentication

According to Eq. (47) in our proposed scheme, the physical context awareness (location in the IoT system) used involves checking the identities of the edge devices seen by the device Ed being authenticated. Thus, if the subset *D*_*ed*_ is valid and does not contain identities of unknown devices, then the location of our device is authenticated.

#### 6.2.7. Prevention against Secrecy Attack

In each session, the session key SK is built using four different random numbers that are generated by Hd, *S*, *T*, and Ed. When an attacker tries to compromise the session key SK, the confidential information of past or future communication sessions can not be compromised. For this reason, the proposed scheme prevents secrecy attacks.

#### 6.2.8. The Property of Identity Anonymity

Two of the essential security properties in the authentication process are anonymity of identity and untraceability. Identity anonymity ensures that the identity of handheld devices is maintained securely so that Hd stays unidentifiable among other devices. Therefore, an attacker is unable to identify the devices' identities. Untraceability, on the other hand, means that the various sessions set up by a specific handheld device are not traceable; so, an attacker cannot relate any sessions to a specific handheld device. These two main security properties were achieved by the use of the emergency practitioner's dynamic identity, where we use a different ID in each session.

#### 6.2.9. Prevention against Cloning Attack

Cloning attack targets unprotected IoT edge devices.  Section 3 discussed incorporating PUF modules in the edge devices which provides a high degree of tamper-resistant unique identity (fingerprint) without incurring extra costs in the area, delay, power, or specialized processing steps. The unique device identity avoids using nonvolatile memory, whose contents can be easily obtained by an unsophisticated attacker. Instead, the device ID is captured in the PUF circuitry which provides a random response with low entropy that imparts sufficient differences between the manufactured edge devices. Therefore, IoT edge devices are immune to cloning attacks.

#### 6.2.10. Prevention against Physical Attack

Physical attack attempts to obtain the secret key of the device knowing that secret keys are typically stored in nonvolatile memories. The IoT edge devices are the most vulnerable to this type of attack since they are usually located in unsecured locations. The PUF response is used to extract the secret key instead of relying on nonvolatile memories.  Section 4.7 discussed how the secret key is extracted from the noisy response of the PUF. Therefore, IoT edge devices are immune to physical attacks.

### 6.3. Performance and Comparative Analysis

In this section, we assess the performance of our proposed scheme in terms of computation costs and storage costs. A comparative analysis of the security features and defense against various attacks is presented.

Our proposed scheme has four entities (Hd, *S*, *T*, and Ed) for which in this subsection, we evaluate storage cost requirements (in bits). SHA-3 is used as an example of the hash function. SHA-3 standard is adapted from the Keccak hash function where four versions of SHA3-224, SHA3-256, SHA3-384, and SHA3-512 are called, depending on the output length n [50]. To evaluate the storage cost requirements, SHA3-384 is utilized where the output of SHA3-384 is 384 bits. In our proposed scheme, Hd stores ID*_hd_* and *V*_*hd*_; therefore, based on SHA3-384, we get *V*_*hd*_ = 384 bits while ID_*hd*_ = 128 bits. As result, the total storage cost requires for Hd is 384 + 128 = 512 bits. Similarly to *S*, ID*_s_*, ID*_hd_*, ID*_t_*, *V*_*hd*_, and *V*_*t*_ are stored by *S*. ID_*s*_ = ID_hd_ = ID_*t*_ = 128 bits, and the storage cost is (2 × 128). As *V*_*hd*_ equal to *V*_*t*_, the storage cost is 2 × 384 when applying SHA3-384. Thus, the total storage cost required for *S* is (2 × 384) + (3 × 128) = 1152 bits. The total storage costs required for *T* is (2 × 384) + (3 × 128) = 1152 bits, as *T* stores ID*_t_*, ID*_s_*, ID_ed_, *V*_*t*_, and *V*_*ed*_ where ID_*t*_ = ID_*s*_ = ID_*ed*_ = 128 bits and *V*_*t*_ = *V*_*ed*_ = 384 bits. The entity of Ed requires 512 bits as the total storage cost. Ed stores ID*_ed_* and *V*_*ed*_ where ID_*ed*_ = 128 bits and *V*_*ed*_ = 384 bits.

The parameters considered for calculating the computation costs are scalar multiplication *T*_*M*_, asymmetric encryption/decryption *T*_*A*_, execute/verify a signature *T*_sign_, symmetric encryption/decryption *T*_*S*_, bilinear pairing *T*_*P*_, and one-way hash function *T*_*H*_. The time required to conduct certain operations is illustrated in Table 2. Due to a negligibly small amount of delay, the computation time of the exclusive-OR (XOR) function is omitted. We used similar experimental values as employed in [51]. Our scheme performs 13 hash invocations and 19 XOR operations, which yields a total computation cost (13 × *T*_*h*_). Therefore, the computation cost of our proposed protocol is (13 × 0.33 ms) = 4.29 ms. A performance costs comparison between our scheme and previous ones is shown in Table 3.

The security features and prevention against various attacks are compared between our scheme and the previous schemes as presented in Table 4. We can claim that our proposed scheme provides more protection against many attack types more than compared schemes.

## 7. Conclusion

A secure transporter healthcare delivery scheme using multifactor authentication of the ambulance is proposed. The transporter could be an ambulance or an air ambulance. We used physical unclonable functions (PUFs) embedded in the ambulance to facilitate authentication and secure key exchange. Formal and informal security analysis was conducted to validate our proposed scheme. The AVISPA tool was used to assure us of the security of the protocol. The performance of our proposed scheme in terms of computation costs and storage cost was assessed compared to the previous proposed schemes. As a result, our proposed scheme shows more protection against many attack types more than compared schemes.

## Figures and Tables

**Table 1:** Notations.

Symbol	Description
*T*	Transporter
*S*	Server
*E*_*d*_	Edge device: sensor or actuator
*H*_*d*_	Handheld device: smartphone, tablet, etc.
Emergency practitioner	Person giving emergency services such as a paramedic, doctor, or a nurse using a handheld device *H*_*d*_
ID*t*	Identity of transporter *T*
ID*s*	Identity of server *S*
ID*_ed_*	Identity of edge device Ed
ID*_hd_*	ID_hd_ Identity of handheld device Hd
*B*_*hd*_	Biometric of handheld device user
DB*_ed_*	CRP dataset associate with built-in PUF of edge device Ed
*K*_*t*_	Symmetric key of transporter *T*
*K*_*s*_	Symmetric key of server *S*
*K*_*ed*_	Symmetric key of edge device Ed
*K*_*hd*_	Symmetric key of handheld device Hd
*k*	Session secret key
*x*	PUF-based secret key
*N*	Nonce
*x* = = *y*	Check equality of *x* and *y*
*Ep* (*k*, *m*)	Symmetric encryption of message m with public key *K*
*Es* (*k*, *m*)	Symmetric encryption of message m with secret key *K*
*Dp* (*k*, *m*)	Symmetric decryption of message m with public key *K*
*Ds* (*k*, *m*)	Symmetric decryption of message m with secret key *K*
*h* (*m*)	Collision-resistant one-way hash function of message *M*
*h* (*k*, *m*)	Collision-resistant one-way cryptographic hash of message *M* using secret key *K*
Request (ID_s_,ID_ed_, *m*)	Request to communication between source ID_s_ and destination ID_ed_ and an encrypted message
*A*⟶*B* : *m*	A sends a message to B through a communication channel
*m*1‖*m*2	Concatenating two messages m^1^ and m^2^
*m*1 ⊕ *m*2	Bitwise XOR operation between m^1^ and m^2^

**Table 2:** The computational time of crypto-operations [51, 52].

Operation	Description	Time (ms)
*T*_*M*_	Scalar multiplication	32.3
*T*_*A*_	Asymmetric encryption/decryption	311.8
*T*_sign_	Execute/verify a signature	322.3
*T*_*S*_	Symmetric encryption/decryption	7.2
*T*_*P*_	Bilinear pairing	31.3
*T*_*H*_	One-way hash function	0.33

**Table 3:** Performance comparison (computation costs).

Scheme (authentication)	Total cost	Time (ms)
Ref. [17]	21*T*_*H*_ + 3*T*_*P*_ + 10*T*_*M*_	196.02
Ref. [16]	17*T*_*H*_ + 5*T*_*S*_	52
Ref. [52]	8*T*_*H*_ + 2*T*_*A*_ + 5*T*_*M*_ + 2*T*_*S*_	37.30
Ours	13*T_H_*	4.29

**Table 4:** A comparison of security-based functionality features.

Feature	Ref. [17]	Ref. [16]	Ref. [52]	Ours
Prevention against reply attack	Yes	Yes	Yes	Yes
Prevention against impersonation attack	Yes	Yes	No	Yes
Prevention against MITM attack	Yes	No	No	Yes
Prevention against confidentiality/privacy attack	No	Yes	Yes	Yes
Prevention against SK guessing attack	Yes	No	Yes	Yes
Location-based authentication	No	No	No	Yes
Prevention against secrecy attack	Yes	Yes	Yes	Yes
The property of identity anonymity	Yes	Yes	Yes	Yes
Prevention against cloning attack	No	No	No	Yes
Mutual authentication	Yes	Yes	Yes	Yes
Prevention against physical attack	No	No	No	Yes
Formal verification (AVISPA)	No	No	Yes	Yes

## Data Availability

The data is available upon author request.
